# Aging and sex differences in salt sensitivity of blood pressure

**DOI:** 10.1042/CS20240788

**Published:** 2025-01-28

**Authors:** Mert Demirci, Jeremiah M. Afolabi, Annet Kirabo

**Affiliations:** 1 Department of Medicine, Division of Nephrology and Hypertension, Vanderbilt University Medical Center, Nashville, TN, U.S.A; 2 Department of Medicine, Division of Clinical Pharmacology, Vanderbilt University Medical Center, Nashville, TN, U.S.A; 3 Department of Molecular Physiology & Biophysics, Vanderbilt University, Nashville, TN, U.S.A; 4 Vanderbilt Center for Immunobiology, Vanderbilt University Medical Center, Nashville, TN, USA; 5 Vanderbilt Institute for Infection, Immunology and Inflammation, Vanderbilt University Medical Center, Nashville, TN, USA; 6 Vanderbilt Institute for Global Health, Vanderbilt University Medical Center, Nashville, TN, USA

**Keywords:** inflammation, salt sensitivity, hypertension, aging, sex differences

## Abstract

Salt sensitivity of blood pressure (SSBP) is a complex physiological trait characterized by changes in blood pressure in response to dietary salt intake. Aging introduces an additional layer of complexity to the pathophysiology of SSBP, with mitochondrial dysfunction, epigenetic modifications, and alterations in gut microbiota emerging as critical factors. Despite advancements in understanding these mechanisms, the processes driving increased salt sensitivity with age and their differential impacts across sexes remain unclear. This review explores the current understanding of salt sensitivity, delving into its underlying mechanisms, the role of inflammation, and the influence of aging and sex differences on these processes. We also aim to provide insights into the multifaceted nature of salt sensitivity and its implications for personalized treatment strategies in hypertension management.

## Introduction

According to the World Health Organization, hypertension remains a significant global health concern, affecting approximately 1.28 billion adults worldwide [1]. This chronic condition is a major risk factor for cardiovascular diseases, including heart failure, stroke, and kidney disease, making it a leading cause of morbidity and mortality [2]. Despite advances in treatment options, the prevalence of hypertension continues to rise, particularly in aging populations and in developing countries, thereby increasing patient mortality [3]. One crucial factor in the development and progression of hypertension is salt sensitivity of blood pressure (SSBP). It is defined as an increased reactivity of one’s blood pressure in response to changes in dietary sodium intake [4]. This physiological trait is not uniform across populations; it varies significantly between individuals and is influenced by factors such as genetics, age, sex, and race [4,5]. Understanding the mechanisms underlying salt sensitivity and how these factors influence it is essential for developing effective prevention and treatment strategies.

The relationship between salt intake and blood pressure has been the subject of extensive research for decades. The INTERSALT study, a landmark investigation involving 52 populations worldwide, demonstrated a significant positive correlation between salt intake and blood pressure [6]. Subsequent studies have further solidified this relationship, leading to global recommendations for reduced salt intake as a public health measure to combat hypertension [7]. However, the response to dietary salt is not uniform across all individuals. While some people experience significant increases in blood pressure with increased salt intake (salt-sensitive), others show little to no change (salt-resistant) [8]. This variability in SSBP has important implications for both the prevention and treatment of hypertension, suggesting that a generalized approach to sodium restriction may not be optimal. In addition to high salt intake, diets rich in ultra-processed foods (UPFs) are highly prevalent in the United States and other industrialized nations and have been found to be associated with a higher incidence of hypertension [9,10]. A prospective cohort study involving nearly 15,000 individuals indicates that those in the highest quartile of UPF consumption have a 24% greater risk of developing chronic kidney disease (CKD) compared with those in the lowest quartile [11]. Taken together, it is plausible to think that the human kidney may not be evolutionarily adapted to handle the elevated levels of salt and UPFs consumed daily. Addressing these dietary patterns is crucial for reducing hypertension-related morbidity and mortality. However, evidence directly linking UPF consumption to the pathogenesis of SSBP remains limited. Age is a critical factor in salt sensitivity. As individuals age, their blood pressure becomes more sensitive to salt intake [12]. This age-related increase, in conjunction with the myriad physiological alterations associated with aging, contributes to the elevated incidence of hypertension among older populations. The mechanisms underlying this age-related change are multifaceted, involving alterations in kidney function, hormonal regulation, and vascular physiology [13]. Equally important are the sex differences observed in salt sensitivity.

This review aims to contribute to the existing body of knowledge by synthesizing the current understanding of SSBP, aging, and sex differences. We also aim to provide updated perspectives on this complex physiological trait. Our goal is to highlight the areas for future research and to inform the development of more personalized and effective strategies for managing hypertension across diverse populations.

## Mechanism of salt sensitivity of blood pressure

SSBP is a complex trait involving multiple physiological systems. The main mechanisms include alterations in renal sodium handling, activation of the renin–angiotensin–aldosterone system (RAAS), and endothelial dysfunction. Changes in the gut and kidney axis, immune system, and sympathetic nervous system also play key roles [14–19].

Renal sodium handling plays a central role in salt sensitivity. In salt-sensitive individuals, an increase in sodium intake leads to an impaired ability of the kidneys to excrete excess sodium, resulting in increased blood volume and, consequently, elevated blood pressure [20]. This process is regulated by the complex interplay of sodium transporters along the nephron, including the sodium-chloride cotransporter (NCC) in the distal convoluted tubule and the epithelial sodium channel (ENaC) in the collecting duct [21]. Research has shown that salt-sensitive individuals may have increased expression or activity of these transporters, leading to enhanced sodium reabsorption [22].

RAAS is another key player in salt sensitivity. Under normal conditions, high salt intake suppresses RAAS activity, promoting sodium excretion. However, in salt-sensitive hypertension, there is often an inappropriate activation of the RAAS, leading to increased sodium retention and vasoconstriction [23]. This paradoxical activation of RAAS in the face of high salt intake may be due to genetic factors or acquired renal injury [24]. Beyond the classic RAAS pathway, studies have identified tissue-specific RAAS in various organs, including the brain and blood vessels, which may contribute to salt sensitivity independently of systemic RAAS [25]. Specifically, Teruya et al. showed that intracerebroventricular (ICV) administration of an angiotensin II receptor antagonist lowered blood pressure in salt-sensitive rats compared with salt-resistant rats [26]. Later, Huang et al. corroborated these findings by demonstrating that, in Dahl salt-sensitive rats, a high-salt diet leads to local synthesis of aldosterone in the hypothalamus. Moreover, ICV administration of an aldosterone synthase inhibitor prevented the salt-induced increase in hypothalamic aldosterone and hypertension associated with a high-salt diet.[27] These findings underscore the role of central RAAS in regulating cardiovascular responses to high salt intake in salt-sensitive models.

Endothelial dysfunction, characterized by reduced nitric oxide bioavailability and increased oxidative stress, is another critical mechanism in salt sensitivity. High salt intake can impair endothelial function, leading to reduced vasodilation and increased vascular resistance [28]. Salt loading has been shown to increase the production of reactive oxygen species (ROS) in the vascular wall, which can scavenge nitric oxide and contribute to endothelial dysfunction [29]. Moreover, salt-sensitive individuals appear to have an exaggerated endothelial response to salt loading, potentially due to genetic factors or pre-existing vascular damage [30].

Sympathetic nervous system activation is another important mechanism in salt-sensitive hypertension. Studies have shown that salt-sensitive individuals exhibit increased sympathetic nervous system activity in response to salt loading, leading to increased heart rate, cardiac output, and peripheral vascular resistance [31]. This heightened sympathetic activity may be due to altered central nervous system processing of sodium signals or changes in baroreceptor sensitivity [32]. Specifically, the central nervous system has specialized regions in the circumventricular region that sense sodium concentrations in the blood and cerebrospinal fluid, such as the subfornical organ and the organum vasculosum of the lamina terminalis. These regions trigger responses to maintain sodium balance through thirst and salt appetite regulation [33].

The role of the immune system in salt sensitivity has gained increasing attention recently. High salt intake has been shown to activate various immune cells, including T cells and macrophages, leading to the production of pro-inflammatory cytokines [34]. These cytokines can contribute to vascular inflammation, oxidative stress, and, ultimately, hypertension. In addition, high salt can also alter the immune cell phenotypes, primarily by driving the pro-inflammatory state by influencing the differentiation and activation of various immune cells, such as the naive CD4+ cells into pro-inflammatory T helper 17 (Th17) cells and macrophages into salt-specific pro-inflammatory state [35] while suppressing anti-inflammatory cell types like M2 macrophages and regulatory T cells (Tregs) [36,37]. The discovery of the isolevuglandins (IsoLGs)–inflammasome pathway, which will be discussed in more detail in the next section, has provided further insights into the link between salt, inflammation, and hypertension.

It is important to note that these mechanisms are intricately linked, contributing to the multifaceted nature of salt sensitivity. For example, endothelial dysfunction can lead to reduced nitric oxide production, which, in turn, can enhance sympathetic nervous system activity [38]. Similarly, the activation of the RAAS can promote inflammation and oxidative stress, further exacerbating endothelial dysfunction [39]. Understanding these mechanisms is crucial for developing targeted therapies for salt-sensitive hypertension. For instance, drugs that target specific sodium transporters in the kidney, such as SGLT2 inhibitors, are particularly effective in SSBP [40]. Similarly, interventions that improve endothelial function or reduce inflammation may also be beneficial in managing SSBP [41].

## Inflammation and salt sensitivity of blood pressure

The role of inflammation in the pathogenesis of hypertension, particularly salt-sensitive hypertension, has gained significant attention in recent years. Emerging evidence suggests that inflammation is not merely a consequence of hypertension but plays a causal role in its development and progression [42]. This section will explore the intricate relationship between inflammation and SSBP.

Chronic low-grade inflammation is a hallmark of hypertension, characterized by increased levels of pro-inflammatory cytokines and infiltration of immune cells into target organs such as the kidneys and blood vessels [43]. Salt-sensitive individuals exhibit an exaggerated inflammatory response to high salt intake, which contributes to the development of hypertension [44]. This heightened inflammatory state is associated with oxidative stress, endothelial dysfunction, and vascular remodeling, all of which contribute to elevated blood pressure [45]. Several mechanisms link salt intake to inflammation. High salt concentrations can directly activate immune cells, including T cells and macrophages, leading to the production of pro-inflammatory cytokines such as interleukin-17 (IL-17) and interferon gamma (IFN-γ) [46]. These cytokines, in turn, promote vascular inflammation and renal sodium retention. Additionally, salt loading has been shown to induce the formation of ROS in various tissues, further exacerbating inflammation and oxidative stress [47].

In recent years, the IsoLGs–inflammasome pathway has also been elucidated, revealing a novel mechanistic link between salt intake, oxidative stress, inflammation, and hypertension [48]. IsoLGs are highly reactive γ-ketoaldehydes formed through the free radical-mediated oxidation of arachidonic acid. These molecules rapidly adduct to proteins, altering their function and triggering inflammatory responses [49]. In the context of salt sensitivity, increased salt intake has been shown to promote the formation of IsoLGs in immune cells, particularly in dendritic cells [50]. The accumulation of IsoLGs activates the NLRP3 inflammasome, a multiprotein complex that plays a central role in innate immunity. Activation of the NLRP3 inflammasome produces pro-inflammatory cytokines, such as IL-1β and IL-18, which contribute to vascular inflammation and oxidative stress [48].

Age-related changes in the immune system, known as immunosenescence, may contribute to the increased prevalence of SSBP in older individuals. Aging is associated with a chronic low-grade inflammatory state, termed ‘inflammaging,’ characterized by elevated levels of pro-inflammatory cytokines and diminished anti-inflammatory mechanisms [51]. Specifically, elevated levels of C-reactive protein (CRP), IL-6, and tumor necrosis factor-alpha (TNF-α) are seen in the blood [52]. Several factors contribute to inflammaging, including cellular senescence (i.e. accumulation of old damaged cells), increased oxidative stress, gut microbiota dysbiosis, and immune system dysregulation [53]. This pro-inflammatory milieu may predispose older individuals to SSBP by amplifying the inflammatory response to salt loading [54].

## Sex differences in salt sensitivity of blood pressure

Sex differences play a significant role in the prevalence and manifestation of salt sensitivity. Premenopausal women generally exhibit lower rates of hypertension diagnosis but are more salt-sensitive compared with age-matched men [55–58]. Moreover, sex-specific analyses reveal that the risk of cardiovascular disease (CVD) begins to rise at lower systolic blood pressure levels in women compared with men, highlighting a gender difference in blood pressure-related cardiovascular risk [59].

While multiple studies have demonstrated the cardioprotective effects of estrogen, the increased SSBP observed in women may also be attributed to non-hormonal factors, such as genetics, epigenetic modifications, lifestyle differences, or environmental influences. Combined with the hormonal variations, these factors could contribute to the complex interplay that affects women’s blood pressure regulation and salt sensitivity. For instance, estrogen has been shown to have multiple beneficial effects on blood pressure regulation. Estrogen enhances nitric oxide production by increasing the expression and activity of endothelial nitric oxide synthase (eNOS), promoting vasodilation and improving vascular function [60]. Research by Scuteri et al. showed that administering estrogen replacement to postmenopausal women decreased salt sensitivity, supporting the notion that estrogen’s protective effects diminish after menopause [56,61]. Additionally, estrogen modulates the RAAS, increasing sodium excretion and reducing vasoconstriction [62]. Evidence suggests that females are more prone to developing vascular dysfunction associated with obesity than males [63]. Studies have shed light on this association by showing that females are more susceptible to leptin-induced aldosterone synthesis, which affects endothelial mineralocorticoid receptor (MR) activity [64]. Additionally, women appear predisposed to endothelial dysfunction through progesterone-driven mechanisms that upregulate MR expression. Faulkner et al. demonstrated that *in vitro* incubation of human umbilical vein endothelial cells with physiological doses of progesterone significantly increased MR protein expression [65]. These findings may explain why aldosterone receptor antagonists are more effective in preventing ventricular remodeling after myocardial infarction in female rats [66]. Furthermore, testosterone has been shown to decrease plasma aldosterone levels by downregulating aldosterone synthase [67]. These hormonal effects and non-hormonal factors contribute to the complex interplay affecting blood pressure regulation and SSBP in women.

**Figure 1:**
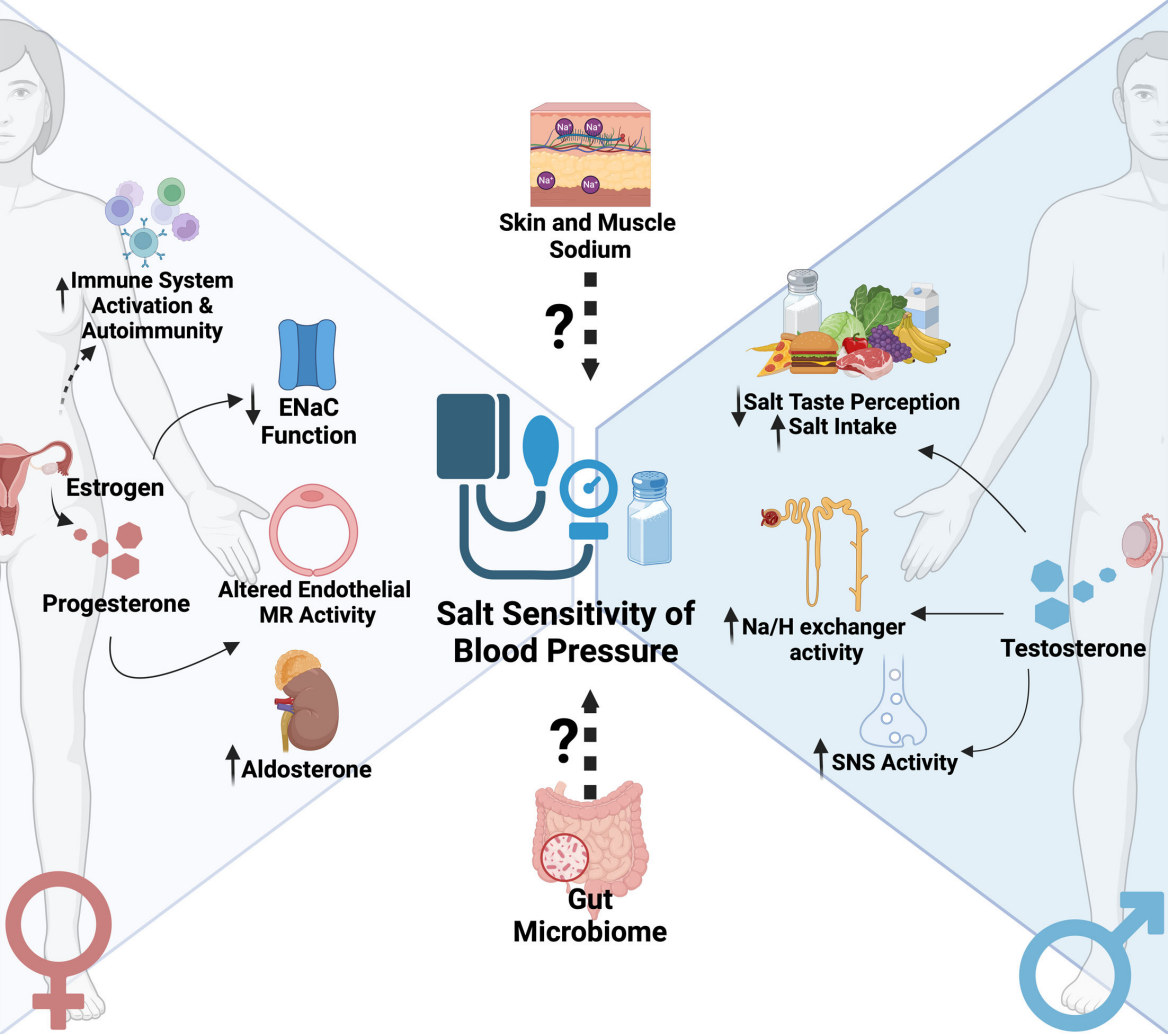
Factors contributing to sex differences in salt sensitivity of blood pressure This figure highlights the potential mechanisms by which sex hormones modulate salt sensitivity of blood pressure. ENaC, epithelial sodium channel; MR, mineralocorticoid receptor; Na/H, sodium-hydrogen; PR, progesterone receptor; SNS, sympathetic nervous system. Figure was created with BioRender.

Recent research has also highlighted the importance of sex differences in immune responses and their potential impact on salt sensitivity (Figure 1). Women generally mount stronger immune responses than men, which can be both beneficial and detrimental depending on the context [68]. This could also explain the striking female prevalence of some autoimmune diseases, with some reports indicating a male-to-female ratio as high as 1:10 [69]. In the case of salt-sensitive hypertension, the stronger immune responses in women may contribute to a more robust inflammatory response against salt-induced damage [70]. In contrast with these findings, estrogen has been shown to suppress the production of pro-inflammatory cytokines and reduce oxidative stress, potentially mitigating the inflammatory responses associated with high salt intake [71].

Studies have shown that testosterone can enhance sodium reabsorption in the kidney, particularly through increased expression and activity of the sodium–hydrogen exchanger (Na/H) in the proximal tubule [72]. Additionally, testosterone has been linked to increased sympathetic nervous system activity, which can contribute to salt-sensitive hypertension [73]. Despite the fact that testosterone increases salt sensitivity, evidence is lacking to explain why women are more salt-sensitive than men in all ethnicities and ages. This discrepancy suggests that sex differences in salt sensitivity are not solely attributable to sex hormones. Genetic factors, including those related to the angiotensin II type 2 receptor (AT2R) gene located on the X chromosome, have been implicated in salt sensitivity and show differential expression between males and females [74,75]. Moreover, the ENaC plays a crucial role in sodium homeostasis and blood pressure regulation, with notable sex differences observed in their expression and function [76]. Females generally exhibit lower ENaC activity than males, which is attributed to the suppressive effects of estrogen on ENaC expression and function [56,77]. Conversely, androgens have been shown to enhance ENaC activity, which may explain the elevated susceptibility to SSBP observed in postmenopausal women, coinciding with a relative increase in androgen levels following the decline of estrogen [78].

As discussed earlier, the gut microbiome, which has been implicated in salt sensitivity, also exhibits sex differences. Studies have shown that the gut microbiota composition differs between males and females, and these differences may influence the metabolic and immune responses to high salt intake [79]. Salt appetite also differs between sexes, with women reported to consume approximately 10% less salt than men [80]. Additionally, men demonstrate lower awareness of their salt consumption compared with women, which may be related to differences in salt taste perception [81]. Further research is needed to understand the implications of salt appetite differences between genders and their potential impact on health outcomes.

As we age, the interplay between sex differences and salt sensitivity becomes even more complex. For example, the effectiveness of certain antihypertensive medications may vary between males and females, and dietary sodium recommendations may need to be tailored based on sex and menopausal status. Hormone replacement therapy in postmenopausal women has shown mixed results in terms of blood pressure management, highlighting the complex nature of sex hormone effects on cardiovascular health [82].

## Effect of aging in salt sensitivity of blood pressure

Aging is associated with hypertension and is a major risk factor for CVD [83]. However, the underlying mechanisms are still not fully understood. The association between salt sensitivity and aging is expected, as age-related vascular dysfunction and reduced kidney function are key factors thought to contribute to the mechanisms underlying SSBP.

It has been established that vascular function plays a more crucial role than renal function in SSBP [16,84]. However, renal function and renal blood flow (RBF) remain important. Reduced RBF is evident in aged individuals, with elderly individuals showing elevated tubular sodium reabsorption due to diminished RBF. RBF remains constant until the fourth decade but declines by about 10% every decade thereafter [85]. Moreover, renal mass, reflecting the amount of healthy glomeruli, decreases from approximately 400 gm at its peak during young adulthood to approximately 300 gm in older individuals [86].

Endothelial function is also affected by age. Aged individuals have increased pulse pressure and pulse wave velocity, reflecting loss of vascular elasticity [87]. Similar to kidney tubular cells, ENaC is abundant in vascular endothelial cells and plays a role in vascular stiffness [88]. Paar et al. investigated endothelial ENaC (EnNaC) function in vascular stiffness and salt sensitivity in the aortas of young and aged mice [89]. They found that older mice had more EnNaC channels in their aortas, leading to increased cortical stiffness under high salt conditions compared with young aortas. Furthermore, the administration of amiloride and spironolactone, which suppresses EnNaC activity and aldosterone action, respectively, was less effective in reducing EnNaC levels in older vs. younger aortas [89]. This may indicate alterations in receptor interactions within the vascular wall, contributing to age-related differences in vascular stiffness. Research has also shown that dietary interventions, such as a low-sodium diet, can improve vascular stiffness independently of blood pressure levels, underscoring the impact of sodium on vascular health [90]. Interestingly, age-standardized brachial-ankle pulse wave velocity is highest in Asian countries, including Japan, China, and Indonesia, where the daily salt consumption ranks highest globally [87,91].

Klotho is a protein that plays a significant role in aging, and it is primarily expressed in the choroid plexus of the brain and distal convoluted tubules in the kidney [92]. Serum soluble Klotho levels decrease with age and have been suggested as a biomarker of aging [93]. Overexpression of the Klotho gene has been shown to extend lifespan, while its knockout accelerates aging-related cognitive decline in mice [94]. Due to these properties of the Klotho gene, it is defined as an ‘anti-aging gene’ and is linked to various age-related diseases such as cancer and dementia [95]. Given that salt sensitivity increases with age, its relationship with Klotho is worth exploring. Citterio et al. investigated this relationship in 673 hypertensive individuals with assessed salt sensitivity phenotypes [96]. They found that a common missense single nucleotide polymorphism in the Klotho gene, rs9536314, was significantly associated with salt sensitivity. Additionally, serum α-Klotho levels were directly correlated with glomerular filtration rate (GFR) values [96]. Notably, patients with CKD exhibit lower serum Klotho levels, suggesting its diagnostic importance and role in kidney aging [97]. Kawarazaki et al. demonstrated that a high-sodium diet induces SSBP in young Klotho heterozygous knockout mice and aged mice. Moreover, Klotho supplementation reversed high salt-induced blood pressure elevation, suggesting that Klotho deficiency contributes to the pathogenesis of SSBP [98].

## Novel insights on aging and salt sensitivity of blood pressure

### Epigenetic modifications

**Figure 2:**
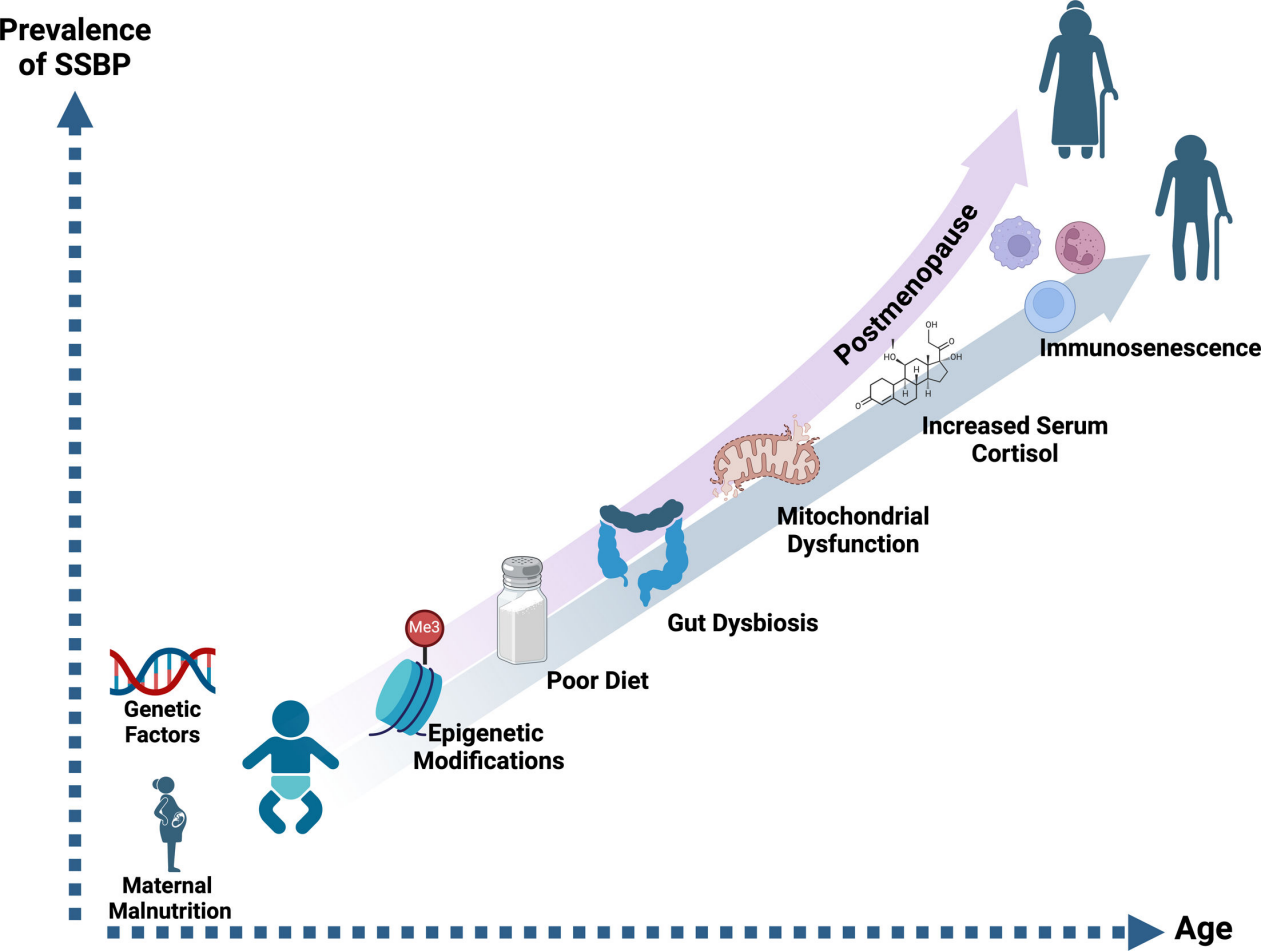
Cumulative influence of factors on the prevalence of salt sensitivity of blood pressure across the lifespan This figure illustrates the cumulative influence of genetic, biological, environmental, and lifestyle factors on the prevalence of salt sensitivity in blood pressure throughout the lifespan in both males and females. The y-axis represents the increasing prevalence of salt-sensitive hypertension, while the x-axis depicts the aging process from infancy through older adulthood. The figure highlights that women are generally more salt-sensitive at all ages, with a notable increase in salt sensitivity during the postmenopausal period. SSBP, salt sensitivity of blood pressure. Figure was created with BioRender.

Epigenetic alterations are hallmarks of the aging process [99]. As individuals age, epigenetic changes naturally accumulate. This process is also referred to as ‘epigenetic drift’ and can impact gene expression in ways that contribute to various age-related conditions, including hypertension. The progressive nature of these epigenetic modifications may exacerbate an individual’s sensitivity to salt, leading to an increased risk of hypertension with advancing age. This accumulation can lead to the dysregulation of genes involved in various critical functions, including metabolism, immune response, and cellular repair mechanisms [100]. Over time, these changes can contribute to the onset and progression of age-related diseases, including hypertension and CVD. While the effects of aging on SSBP are significant, factors before birth, such as prenatal epigenetic modifications, also play a crucial role in determining salt sensitivity (Figure 2). Epigenetic studies have shown that epigenetic modifications can affect the RAAS pathway and renal sodium handling. Maternal malnutrition has been shown to upregulate type 1A angiotensin II receptor (AT1AR) activity in the hypothalamus of the offspring via aberrant DNA methylation, a process linked to decreased 11β-HSD2 levels and excess glucocorticoid levels in the fetus [101]. This, in turn, leads to increased baseline AT1AR receptor activity and renal sympathetic activity, contributing to the postnatal development of SSBP [102]. This mechanism has been further supported by different studies in murine models, where genetic deletion of AT1AR or kidney denervation prevents the development of hypertension in the offspring of pregnant mice and rats fed a low-protein diet.

### Hypertensive disorders of pregnancy and SSBP

In aging women, pregnancy is one of the most significant life-changing events, leading to profound physiological changes in the body. Preeclampsia, a condition thought to stem from endothelial dysfunction and impaired regulation of nitric oxide (NO), has been widely studied for its potential connection to future vascular diseases. During pregnancy, sodium handling and sodium balance undergo significant changes, and hypertensive disorders in pregnancy may influence salt sensitivity, potentially impacting long-term cardiovascular mortality. Indeed, many studies have investigated the link between preeclampsia and future vascular diseases, as the effect of preeclampsia is not only limited to the pregnancy period. Patients who had hypertensive disorders of pregnancy have an increased risk of CVD risk and mortality in later life [103–105]. Martillotti et al. showed that premenopausal women with a history of severe preeclampsia are more likely to be salt-sensitive compared with their salt-resistant counterparts without a history of preeclampsia [106]. Although the retrospective design of the study does not establish causality, it suggests that the salt-sensitive group may have already been predisposed to salt sensitivity during pregnancy. Preeclampsia shares similar racial disparities with salt-sensitive hypertension, being more prevalent among Black women and women of South Asian origin [107]. The impact of preeclampsia on salt sensitivity goes beyond just the mother. Yeung et al. have demonstrated that baboon offspring with preeclampsia also show heightened salt sensitivity, characterized by increased systolic blood pressure and higher aldosterone levels following salt intake [108]. This suggests that fetal programming could play a role in the long-term cardiovascular risks faced by these offspring.

### Mitochondrial dysfunction

One of the unexplored areas within hypertension research is the relationship between mitochondria and SSBP. The role of mitochondrial dysfunction in biological aging is a well-established concept, known as the mitochondrial theory of aging [109]. This theory posits that ROS damage accumulates in mitochondria over time, leading to a decline in cellular function and contributing to the aging process and the onset of various age-related chronic diseases. Indeed, research has shown that mitochondrial dysfunction is present in a wide range of age-related diseases, including, but not limited to, CKD and congestive heart failure [110,111]. Notably, patients with these chronic diseases exhibit accelerated mitochondrial dysfunction compared with their age-matched healthy counterparts, with degree of this dysfunction often preceding the deterioration during the course of the disease [112,113].

Growing bodies of evidence show that the structure and function of mitochondria can be detrimentally affected by a high sodium environment [114]. Specifically, the increase in total body sodium increases intracellular sodium, leading to the activation of the Na^+^/Ca^++^ exchanger on the mitochondrial membrane, loss of calcium, and increases in Na^+^ inside the mitochondria [115]. Since calcium is a crucial molecule for mitochondrial metabolism, this cascade leads to detrimental effects on the mitochondria. Moreover, increased mitochondrial Na^+^ itself inhibits ATP production via inhibiting complex II/III in the mitochondria inner membrane [116]. High sodium levels have been shown to impact the main mitochondrial deacetylase Sirtuin 3 [117]. Specifically, elevated sodium reduces Sirtuin 3 expression by epigenetic modification, which plays a crucial role as a metabolic sensor in regulating energy metabolism, mitochondrial biogenesis, apoptosis, autophagy, and redox metabolism in a coordinated manner [118]. Sirtuin 3 levels decrease by age with levels decreasing approximately by 40% at the age of 65 [119]. Restoring Sirtuin 3 levels by resveratrol supplementation improved vascular dysfunction and lowered hypertension in hypertensive rats [120].

As individuals age, there is a noticeable increase in the incidence of mitochondrial dysfunction, elevated tissue sodium levels, hypertension, and salt sensitivity. Rather than accepting these changes as an inevitable part of aging, it is worth exploring whether the interplay between mitochondrial dysfunction and elevated tissue sodium levels could be a driving factor or a result of the increased prevalence of hypertension and salt sensitivity in older adults.

### Effect of gut health

Being the first and main site for salt absorption, the role of intestinal mucosa and microbiota in the gut has earned significant attention in extrarenal mechanisms of SSBP, as well as in healthy aging [121]. More than a thousand different bacteria species reside in the human gut [122]. This bacterial population is highly dynamic, with their biodiversity changing with age, sex, body-mass index, environmental factors, medication use, and dietary habits [123–125]. Studies have shown that the influence of gut microbiota extends beyond their well-known roles in vitamin and essential amino acid production. These microbes also produce short-chain fatty acids (SCFAs), which play a key role in modulating immune cell function and enterocyte metabolism [126–128]. SCFAs exert their effects by binding to specific G-protein-coupled receptors (GPCRs), known as metabolite-sensing GPCRs, particularly through GPR43, GPR41, and GPR109A [128,129].

An expanding body of research from human and animal studies underscores the critical role of gut microbiota in blood pressure regulation [130]. Studies involving fecal microbiota transplantation (FMT) have provided compelling evidence that transferring gut microbiota from hypertensive humans to germ-free normotensive mice induces a hypertensive response in the recipients [131]. In contrast, germ-free mice that receive FMT from normotensive individuals maintain normal blood pressure levels, highlighting the direct influence of gut microbiota on regulating blood pressure. This demonstrates the profound impact of gut microbiota composition on cardiovascular health and the potential for gut-targeted therapies in managing hypertension [131].

Recent reports have shown that dysbiosis is associated with aging and salt intake [132]. In aged individuals, an increase in tissue sodium levels and sodium consumption can alter the function and composition of gut microbiota [133]. Long-term salt consumption can disrupt the balance of healthy gut microbiota and alter the production of essential bacterial metabolites [134,135]. This dysbiosis-induced inflammation may lead to vascular and renal dysfunction, further contributing to the development and exacerbation of hypertension [134,136].

The research conducted by Ferguson et al. examined the variations in microbiota among individuals categorized by their salt intake [135]. Their results showed that individuals with high salt consumption exhibited higher systolic and diastolic blood pressures, with notable differences in microbiota compared with those with normal salt intake. Furthermore, histological analysis of the intestinal wall revealed increased IsoLG staining intensity and greater infiltration of T cells and macrophages in hypertensive individuals compared with their normotensive counterparts [135]. *Lactobacillus* strains have been shown to help restore gut microbiota balance, improve oxidative stress and inflammation, and potentially enhance longevity [137,138]. In mice, a high-salt diet leads to a depletion in *Lactobacillus murinus* and a significant increase in Th17 cells, which are recognized to play a role in SSBP [139]. Dendritic cells from both aged and gut-dysbiotic young mice have failed to induce regulatory T cells (Tregs), leading to the overactivation of CD4+T cells, a phenomenon that is not present in healthy young mice [140]. This loss of immune tolerance was associated with the overactivation of the NF-κB pathway, an increase in pro-inflammatory molecules, and a decline in anti-inflammatory factors [140]. Interestingly, the study found that replenishing the gut of old mice with *Lactobacillus plantarum* restored the tolerogenic function of dendritic cells, effectively rewiring inflammatory and metabolic pathways [140]. This suggests that maintaining or replenishing beneficial gut bacteria, such as *Lactobacillus* species, could be crucial in managing hypertension and reducing age-related effects, including cognitive decline and other conditions associated with aging [141]. While therapies targeting a healthy gut microbiome are currently being explored, studies investigating dietary interventions to promote healthy aging have generally been limited by small sample sizes and short follow-up periods, as discussed in detail in other reviews [124]. Additionally, most research has utilized probiotic supplementation or dietary patterns such as the Mediterranean diet. It is important to note that, while the Mediterranean diet is widely regarded as health-promoting due to its low levels of processed foods and high fiber content, it is not specifically sodium-restricted [142]. Consequently, sodium intake in these diets often exceeds recommended levels and the effect of low sodium intake on mitigating age-related dysbiosis is still an open question. Additional research is needed to determine whether these interventions can effectively mitigate the deleterious effects of SSBP and aging.

## Conclusion

Aging is an inevitable process for all individuals; however, not every individual suffers from the deleterious effects of aging. Advancements in aging research and scientific breakthroughs can mitigate the impact of aging by addressing age-related changes and ultimately improving individuals' quality of life. In the light of the ongoing studies that continue to elucidate the detrimental effects of excessive salt consumption, the World Health Organization’s recommendation of limiting sodium intake to less than 2 grams per day remains critically important [143,144]. However, it is important to note that current research has not yet provided clear evidence on whether extremely low levels of salt consumption can reverse these adverse effects. Therefore, while reducing salt intake is crucial, more research is needed to determine the full impact of such dietary changes on health outcomes. New insights into the role of gut microbiota, mitochondrial dysfunction, and epigenetic modifications add another layer of complexity to this concept. Understanding the intricate interactions between these pathways, aging, and sodium intake is essential for developing targeted interventions that can unveil more effective and personalized treatment strategies, ultimately improving health outcomes in the aging population.

## Abbreviations

AT2R: angiotensin II type 2 receptor
CKD: chronic kidney disease
CRP: C-reactive protein
ENaC: epithelial sodium channel
GFR: glomerular filtration rate
ICV: intracerebroventricular
IFN-γ: interferon gamma
IL-17: interleukin-17
IsoLGs: isolevuglandins
MR: mineralocorticoid receptor
NCC: sodium-chloride cotransporter
NLRP3: NOD-like receptor family, pyrin domain containing 3
NO: nitric oxide
RAAS: renin-–angiotensin-–aldosterone system
RBF: renal blood flow
ROS: reactive oxygen species
SCFAs: short-chain fatty acids
SSBP: salt sensitivity of blood pressure
TNF-α: tumor necrosis factor-alpha
Th17: T helper 17
Tregs: regulatory T cells
UPFs: ultra-processed foods
eNOS: endothelial nitric oxide synthase
